# Effect of Preharvest Ethephon Application on Selected Biochemical Components and Polyphenol Oxidase Activity in Macadamia Nuts

**DOI:** 10.3390/horticulturae9101101

**Published:** 2023-10-04

**Authors:** Noluthando Noxolo Aruwajoye, Asanda Mditshwa, Lembe Samukelo Magwaza, Mjabuliseni Simon Cloapas Ngidi, Samson Zeray Tesfay

**Affiliations:** 1 Discipline of Crop and Horticultural Science, School of Agricultural, Earth and Environmental Sciences, University of KwaZulu-Natal, Private Bag X01, Scottsville, Pietermaritzburg 3209, South Africa; 2 Plant Science Laboratory, Cranfield University, Bedfordshire MK43 0AL, UK; 3 Department of Agricultural Extension and Rural Resource Management, School of Agricultural, Earth and Environmental Sciences, College of Agriculture, Engineering and Science, University of KwaZulu-Natal, Private Bag X01, Scottsville, Pietermaritzburg 3201, South Africa; 4 Centre for Transformative Agricultural and Food Systems, School of Agricultural, Earth and Environmental Sciences, College of Agriculture, Engineering and Science, University of KwaZulu-Natal, Private Bag X01, Scottsville, Pietermaritzburg 3201, South Africa

**Keywords:** ethephon, preharvest, abscission, macadamia nuts, postharvest, quality, nutrition

## Abstract

Ethephon is a plant growth regulator that triggers diverse responses in plants, such as fruit ripening, leaf senescence, hull senescence, stem elongation, and nut abscission. This study examined how the preharvest application of ethephon 480 SL® affects selected biochemical components and polyphenol oxidase (PPO) activity in two macadamia nut cultivars: ‘788’ and ‘Beaumont’. Ethephon was applied to the trees via a Cima mist blower at rates of 13.33 mL/L per hectare for the ‘788’ cultivar and 16.67 mL/L per hectare for the ‘Beaumont’. Following harvest, the nuts were stored at 25 °C for 72 days, and samples were taken at 18-day intervals. Standard procedures were used to assess the following: total phenolics, total flavonoids, 2,2,-diphenyl-1-picrylhydrazyl (DPPH) assay, Ferric reducing ability power (FRAP) assay, sucrose, total protein, and PPO activities. This evaluation was carried out across a total of four treatments: ethephon-treated nuts from the orchard floor (ED), ethephon-treated nuts from the tree (ET), untreated nuts from the orchard floor (CD), and untreated nuts from the tree (CT). The evaluation’s outcomes were analyzed using a principal component analysis (PCA), a correlation matrix heat map (CMHM), and a graphical assessment. The results unveiled significant correlations and associations among the assessed parameters. The correlation matrix heat map analysis highlighted a strong positive correlation (0.97) between the sucrose and the PPO activity in the ‘Beaumont’ cultivar, supported by the PCA analysis identifying the ED treatment as the most influential. At the storage period’s conclusion, the ED treatment had the highest sucrose content (18.63 mg/g) and polyphenol oxidase activity (1.06 U g^−1^). In the ‘788’ cultivar, a close relationship emerged between the phenolic content, the PPO activity, and the Ferric reducing antioxidant power (FRAP)’s antioxidant activity. Consistently, the CT treatment (untreated nuts) demonstrated positive correlations with several key parameters in both cultivars, displaying heightened phenolic content and antioxidant activities. Consequently, our findings indicate that the CT treatment, involving tree-harvested nuts without ethephon application, could be the preferred option for sustaining macadamia nuts’ quality and shelf life compared to other methods. Moreover, our study underscores the significance of proper storage conditions for maintaining the desired biochemical parameters of macadamia nuts. By comprehending the effects of distinct treatments and harvesting techniques, producers and processors can devise strategies to optimize storage conditions and uphold macadamia nut quality.

## 1 Introduction

The cultivation of *Macadamia integrifolia* nuts has gained immense global popularity due to their unique taste, flavor, and nutritional value [1–4]. Macadamia nuts are also well sought-after due to their positive health attributes, with the nuts containing an average of 8% protein and up to eight essential amino acids [3,5,6]. Therefore, macadamia nuts serve as an alternative source of these essential building blocks for optimal health. Furthermore, macadamia nuts are naturally rich in fats, which comprise up to 75% of their total composition [7,8]. Among these fats, approximately 59% are in the form of monounsaturated fat [7]. Nuts are also a source of vitamin B6 and minerals like manganese, iron, and magnesium [9]. Additionally, they contain phytochemicals such as flavonoids and phenolics that further enhance their nutritional profile [10].

Despite these remarkable attributes, the manual harvesting of macadamia nuts is a costly endeavor, requiring significant investments in terms of labor, time, and resources [11]. Therefore, to mitigate such challenges, growers often employ ethephon. Ethephon has been utilized in numerous studies, demonstrating its capacity to induce various plant responses, including fruit ripening, leaf aging, hull aging, stem elongation, and nut detachment [12,13]. When sprayed on macadamia trees, ethephon stimulates the abscission of mature nuts, thus promoting a uniform nut drop. As a result, the cost of multiple harvest periods and the labor required during the harvesting are reduced [11]. The detachment or shedding of plant organs is regulated through a sequence of complex structural, physiological, biochemical, and molecular changes [14]. These changes directly influence the biochemical composition of the organs, including the nuts. Ethephon decomposes into ethylene, a compound recognized for its substantial involvement in various physiological functions within plants [12]. Notably, ethylene has been reported to trigger the expression of plant defense-related proteins [15]. Wang et al. [16] reported that ethephon application in cotton plants resulted in the acceleration of protein degradation in the boll shell and the transfer of assimilated substances [16]. This suggests that ethephon can potentially influence protein degradation and substance transfer in plant structures. Understanding this relationship is essential for obtaining insights into the effects of ethylene on the ripening processes and the resulting changes in biochemical composition. Consequently, the use of ethephon increases the level of ethylene in plant cells, thereby regulating various physiological functions, and can potentially trigger a wide range of physiological mechanisms [17]. The specific effects of this plant growth regulator on the biochemical components and enzymatic activities, such as polyphenol oxidase, of macadamia nuts remain poorly understood. Addressing these knowledge gaps is essential for advancing our understanding of the impact of ethephon on the biochemical composition and polyphenol oxidase activity of macadamia nuts.

Ethephon has been previously used to induce the abscission of various other crops such as peach, table grapes, and litchi [18–20]. Research findings showed that foliar spray of ethephon increases sugar accumulation in sugarcane [21]. Further research conducted by [22] has confirmed that the application of ethylene promotes increased activity in nutrient sinks, resulting in enhanced sucrose accumulation in genotypes naturally low in sugar content. This finding suggests the potential for cultivars to exhibit different responses to ethephon. Additionally, ref. [23] reported that the application of ethephon led to a reduction in the total anthocyanin, total phenol, soluble solids content, and antioxidant capacity when compared to the control group. These findings imply that ethephon application may have an influence on the biochemical composition of macadamia nuts, particularly regarding their sugar content.

Despite the widespread use of ethephon by macadamia growers and the extensive research conducted on the topic [24–27], there remains a notable gap in comprehensive research regarding the postharvest impact of ethephon on the biochemical composition and polyphenol oxidase activity of macadamia nuts. Therefore, the objective of this study is to assess the effect of preharvest foliar spray of ethephon on the postharvest, specifically on the total phenolic content, total flavonoid content, antioxidant capacities (Ferric reducing antioxidant power and 2,2-diphenyl-1-picrylhydrazyl) and polyphenol oxidase of two distinct macadamia nut cultivars during the storage phase. Additionally, this study aims to evaluate the variation in the biochemical composition and polyphenol oxidase activity between the nuts harvested directly from the tree and those collected from the ground. By addressing these research gaps, this study seeks to provide valuable insights into the postharvest effects of ethephon on macadamia nut quality and shed light on the differences between tree-harvested and ground-harvested nuts.

## 2 Materials and Methods

### 2.1 Ethephon Spray and Concentration

Macadamia nut cultivars ‘Beaumont’ and ‘788’ were harvested from the Fyvie estates, on uMlaas Road, Camperdown, KwaZulu-Natal, South Africa (latitude: 29°47′50.3″ S.; longitude: 30°27′54″ E.). Ethephon 480 SL®, a plant growth regulator, was used to promote nut abscission in the macadamia trees [28]. The ‘788’ macadamia trees were treated with a dosage of 13.33 mL/L per hectare, while the ‘Beaumont’ macadamia trees received a dosage of 16.67 mL/L per hectare, both administered using a Cima mist blower, to promote nut abscission. The application of ethephon targeted physiologically mature nuts, and the nuts that naturally detached and fell to the ground were considered successfully abscised nuts.

### 2.2 Nut Collection and Preparation

The study involved collecting nuts from two distinct cultivars: the ‘Beaumont’ and the ‘788’. For each cultivar, the harvested nuts included those fallen from both the treated and untreated trees, as well as those directly picked from both the treated and untreated trees. After collection, a sorting process was used to categorize the nuts into four groups based on their treatment: (CD) for the nuts that had naturally fallen from the control trees; (CT) for the nuts that had been manually picked from the control trees; (ED) for the nuts that had naturally fallen from the ethephon-treated trees; and (ET) for the nuts that had been manually picked from the ethephon-treated trees. Following the harvesting, nuts in their husks underwent mechanical dehusking within 24 h. Subsequently, the dehusked nuts were weighed and subjected to a controlled drying process. The drying procedure followed a specific temperature regimen: 35 °C on the first day, 38 °C on the second day, and 50 °C on the third day. This temperature sequence was adopted in alignment with [29], with minor modifications incorporated to enhance kernel recovery and overall kernel quality.

### 2.3 Storage Conditions

In this research study, a total of 600 macadamia kernels were utilized, with 300 kernels assigned to each of the two cultivars, namely the ‘788’ and the ‘Beaumont’. Within each cultivar, these kernels were further divided into four groups: 75 kernels from ET, 75 kernels from ED, 75 kernels from CT, and 75 kernels from CD. These kernels were then organized into three replicates, each comprising five kernels, and securely enclosed in polythene bags. The bags containing the kernels were stored at a temperature of 25 °C for a maximum of 72 days, simulating the conditions that accelerate shelf-life studies. Sampling events occurred at specific intervals of 0, 36, 54, and 72 days to monitor biochemical changes during storage. Following each sampling event, the macadamia nuts were preserved at −20 °C to maintain the quality of the samples for subsequent analysis.

### 2.4 Extraction and Determination of Total Polyphenols Content and Flavonoids

The freeze-dried nuts were blended into powder and the extraction was carried out using a method described by [30] with slight modifications. Briefly, 1 g of each sample was suspended in 10 mL of 80% methanol and continuously shaken for 2 h. Thereafter, the samples were filtered through a Whatman® no. 1 and were then vacuum evaporated. After evaporation, the extracts were re-suspended in 2 mL of 80% methanol and finally passed through a 0.45 μm nylon filter to clear all the remaining particles. The extract was assayed for its antioxidant activity, total phenolic content (TPC), and total flavonoid content (TFC).

#### 2.4.1 Quantification of Total Phenolic Content

The total phenolic content was analyzed as described by [31] with modifications. A sample extract of 100 uL was mixed with 0.2 mL of Folin–Ciocalteu reagent and 1 mL of distilled water, and the solution was allowed to shake for 3 min at room temperature. There-after, 1 mL of 20% sodium carbonate was added to the mixture. The total phenolic content was quantified after 90 min of incubation at room temperature (25 °C). The absorbance of the solution was measured at 765 nm using a Shimadzu US-VIS spectrophotometer. A standard curve of gallic acid was used for the TPC’s quantification. The results were expressed as mg of gallic acid equivalents (GAE) per g of dry weight (DW). The analysis was conducted in triplicate.

#### 2.4.2 Quantification of Total Flavonoid Content

The total flavonoids were quantified following a method described by [32] using Aluminum Chloride (AlCl_3_). A sample extract of 100 μL was added into a cuvette followed by an addition of 200 μL of water followed by 30 μL of 5% NaNO_2_, and the mixture was allowed to stand at room temperature (25 °C) for 5 min. Subsequently, 30 μL of 10% AlCl_3_ was added; the mixture was incubated for 6 min, and, thereafter, 200 μL of 1 mM NaOH was added to the solution. The absorbance of the reaction was read at 510 nm against methanol used as a blank. A standard curve of quercetin was used for the TFC’s quantification. The results were expressed as mg of quercetin equivalents per g of dry weight (DW). The analysis was conducted in triplicate.

#### 2.4.3 2,2,-Diphenyl-1-picrylhydrazyl (DPPH) Assay

The free radical scavenging activity using the DPPH assay was determined following the method described by [33] with slight modifications. A stock solution of 2,2,-diphenyl-1-picrylhydrazyl at a concentration of 0.1 mM was prepared using methanol and was stored at −20 °C until further use. For the assay, 20 μL of the kernel extract was added to a cuvette, followed by the addition of 980 μL of methanol. Subsequently, 1 mL of the DPPH solution was added to the mixture. The solution was then incubated in the dark for 1 h. After incubation, the change in absorbance was measured at 517 nm under dim light using a Shimadzu US-VIS spectrophotometer. The analysis was conducted in triplicate.

The inhibition percentage was calculated using Equation (1):

(1) $$\text{DPPH}\;\text{inhibition}\frac{({\text{Abs}}_{\text{control}}-\left({\text{Abs}}_{\text{Sample}}\right)}{\left({\text{Abs}}_{\text{Control}\;}\right)}\times 100$$

where Abs _control_ is the absorption of the DPPH radical and methanol, and Abs _sample_ is the absorption of the DPPH radical and sample extract.

#### 2.4.4 Ferric Reducing Ability Power (FRAP) Assay

The Ferric reducing ability power (FRAP) assay was determined according to [34]. The FRAP reagent was prepared by mixing 60 mL of acetate buffer (300 mmol/L. pH 3.6), 6 mL ferric chloride solution (20 mmol/L), and 6 mL of 10 mmol/L 2,4,6-tripyridyl-s-triazine (TPTZ) solution (TPTZ in 40 mM HCl) in a ratio of 10:1:1. The mixture was allowed to stand in a water bath at 37 °C for 15 min. The FRAP reagent (600 μL) was added to 80 μL of extract and 1 mL of water. A reagent blank was prepared as above, with 80 μL water added instead of the test sample. The change in absorbance was recorded at 593 nm using a spectrophotometer. The analysis was conducted in triplicate. A standard solution of ferrous sulphate was prepared. The total antioxidant capacity was expressed as μmoles Fe ^2+^/g of dry weight (DW).

### 2.5 Sucrose Analysis

The sugar content was determined using an HPLC-refractive-index detector (RID) based on the method outlined by [35] with slight modifications. Freeze-dried material weighing 0.10 g was mixed with 10 mL of 80% (*v/v*) ethanol and homogenized for 1 min. The mixture was then incubated in an 80 °C water bath for 60 min to extract the soluble sugars. Following incubation, the mixture was refrigerated at 4 °C overnight. The samples were subsequently filtered through glass wool and dried in a vacuum concentrator. The dried samples were then re-suspended in 2 mL of ultra-pure water, filtered through a 0.45 μm nylon filter, and analyzed using an isocratic HPLC system equipped with a RID. A Phenomenex® column (Rezex RCM–Monosaccharide) was used for the analysis. The concentration of sucrose was determined by comparing the samples to the known sucrose standard.

### 2.6 Protein Extraction

Freeze-dried nuts were ground into a powder, and the extraction was performed using a modified method based on [36]. In this process, 0.5 g of each sample was suspended in 5 mL of TRIS buffer (100 mM, pH 7.5). Subsequently, the samples were centrifuged at 10,000 RPM for 15 min at 4 °C. The supernatant was then filtered through glass wool to remove any remaining floating particles, and the extract was stored for further protein analysis.

#### 2.6.1 Protein Assay Using the Bradford Method

The protein assay was conducted following the procedure described by [36] with slight modifications. To begin, 400 μL of the Bradford reagent was added to 40 μL of the protein extract. The mixture was incubated for 5 min and then read at an absorbance of 590 nm. The results were expressed as micrograms of bovine serum albumin (BSA) per gram of dry weight (DW). The analysis was performed in triplicates.

#### 2.6.2 Quantification of Polyphenol Oxidase

The activity of polyphenol oxidase (PPO) was determined following the method described by [29], which is based on the assay previously outlined by [37]. To conduct the assay, a 100 mL sample of extracted protein was mixed with a combination of 1.45 mL of 20 mM 3-methyl-catechol and 1.45 mL of 10 mM acetate buffer (pH 5.0). The total PPO activity was measured using a spectrophotometer at a wavelength of 420 nm and was expressed as U g^−1^ of sample dry weight.

### 2.7 Data Analysis

An analysis of variance (ANOVA) was carried out using GenStat® 20th Edition (VSN International Ltd., Hertfordshire, UK). The means were separated using the least significant difference (LSD) test at 5% levels of significance. A principal component analysis (PCA), biplot diagrams, and a correlation matrix heatmap were deployed to facilitate the comprehensive identification of the effect of ethephon on macadamia nuts’ postharvest quality using Python programming.

## 3 Results and Discussion

### 3.1 Total Phenolic Contents (TPC) and Total Flavonoid Contents (TFC)

**Figure 1:**
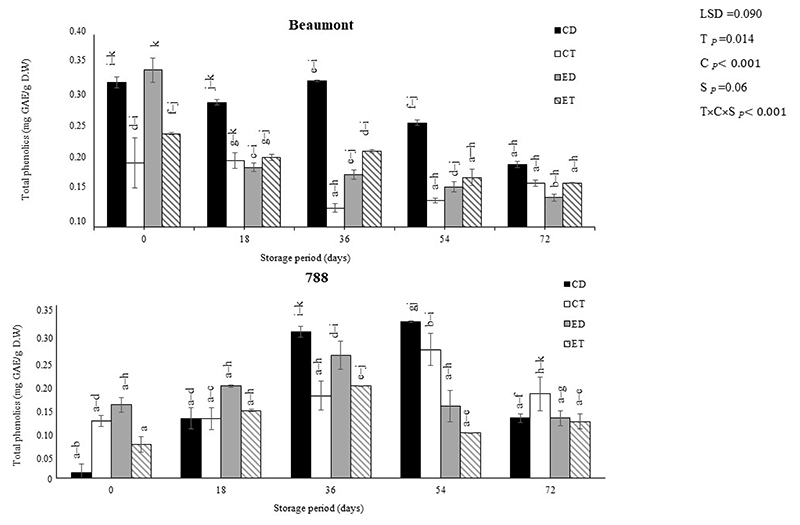
The effect of ethephon on the total phenolic content of macadamia nuts during storage. Data are expressed as mean ± standard errors of three replicates. Vertical bars represent the standard errors of the means. CD represents nuts that dropped from the control tree; CT are nuts that were directly picked from the control tree; ED represents nuts that dropped from the ethephon-treated trees; and ET represents nuts that were picked directly from the ethephon-treated trees. Lowercase letters (a−k) indicate differences between the treatments during storage. T represents treatment (ethephon or control); C stands for cultivar; and S represents storage duration in days.

**Figure 2:**
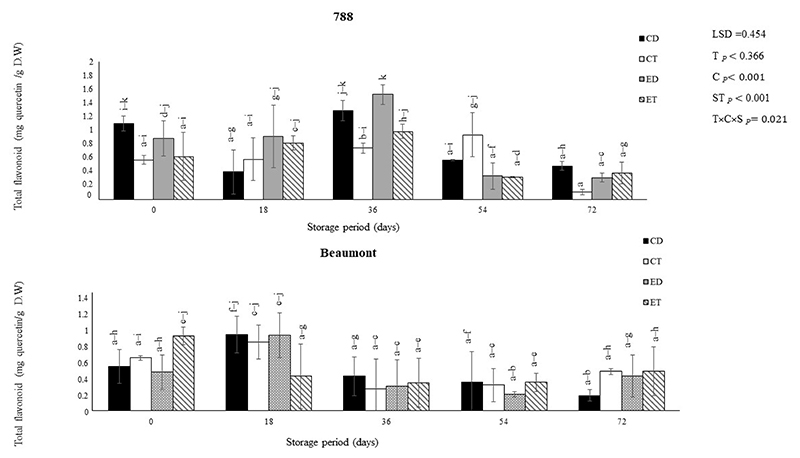
The effect of ethephon on the total flavonoid content of macadamia nuts during storage. Data are expressed as mean ± standard errors of three replicates. Vertical bars represent the standard errors of the means. CD represents nuts that dropped from the control tree; CT are nuts that were directly picked from the control tree; ED represents nuts that dropped from the ethephon-treated trees; and ET represents nuts that were picked directly from the ethephon-treated trees. Lowercase letters (a−k) indicate differences between the treatments during storage. T represents treatment (ethephon or control); C stands for cultivar; and S represents storage duration in days.

Flavonoids and phenolics are polyphenolic compounds that have numerous health benefits and occur naturally in a wide range of foods, including macadamia nuts [3,30]. The impact of different treatments (CD, CT, ED, and ET) on the TPC and TFC of macadamia nuts (‘Beaumont’ and ‘788’) stored at 25 °C is depicted in Figures 1 and 2, respectively. For the ‘Beaumont’ nuts, the ET decreased over time but had the highest phenolic treatment among all treatments (except for the CD) on day 0. The decrease in the TPC in the ET and ED nuts could be attributed to the influence of the ethylene released from the ethephon, which can induce enzymatic degradation of phenolic compounds (Figure 5) [38,39]. On days 0 and 18, the CD nuts maintained a higher TPC compared to the ET nuts. This observation suggests that the CD treatment may have promoted the accumulation or preservation of phenolic compounds during the initial stages of storage in the control treatment, resulting in an increased TPC. Conversely, for the ‘788’ nuts, all treatments generally resulted in an increase in TPC up to day 36 and then a decrease. The CT treatment had the highest TPC (0.25 mg GAE/g) at the end of the storage period.

### 3.2 Antioxidant Activity (DPPH and FRAP)

**Figure 3:**
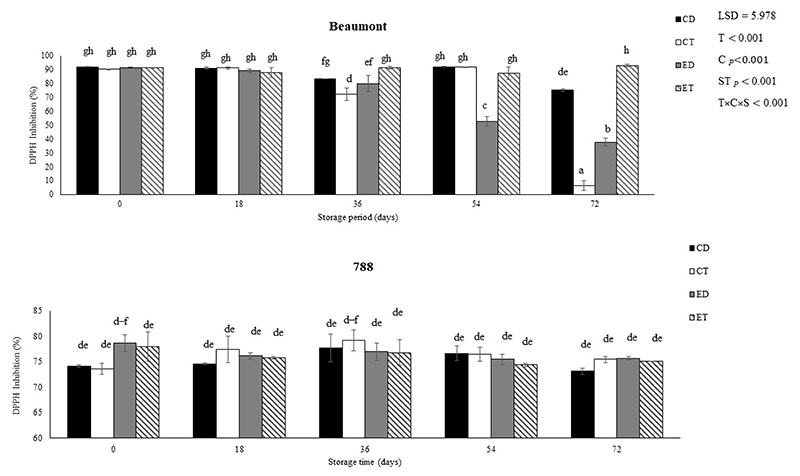
The effect of ethephon application on the DPPH of macadamia nuts during storage. Data are expressed as mean ± standard errors of three replicates. Vertical bars represent the standard errors of the means. CD represents nuts that dropped from the control tree; CT are nuts that were directly picked from the control tree; ED represents nuts that dropped from the ethephon-treated trees; and ET represents nuts that were picked directly from the ethephon-treated trees. Lowercase letters (a−h) indicate differences between the treatments during storage. T represents treatment (ethephon or control); C stands for cultivar; and S represents storage duration in days.

**Figure 4:**
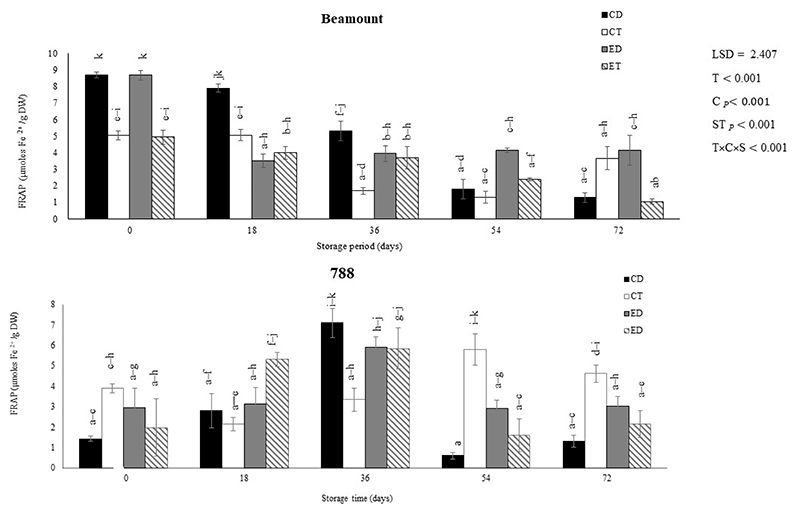
The effect of ethephon application on the FRAP of macadamia nuts during storage. Data are expressed as mean ± standard errors of three replicates. Vertical bars represent the standard errors of the means. CD represents nuts that dropped from the control tree; CT are nuts that were directly picked from the control tree; ED represents nuts that dropped from the ethephon-treated trees; and ET represents nuts that were picked directly from the ethephon-treated trees. Lowercase letters (a−k) indicate differences between the treatments during storage. T represents treatment (ethephon or control); C stands for cultivar; and S represents storage duration in days.

The assessment of the DPPH and FRAP radical scavenging rates is a common method used to evaluate the antioxidant capacity of fruits and vegetables [40]. The impact of different treatments (CD, CT, ED, and ET) on the DPPH and the FRAP of macadamia nuts (‘Beaumont’ and ‘788’) stored at 25 °C is depicted in Figures 3 and 4, respectively. For the cultivar ‘Beaumont’, the DPPH inhibition percentage (%) was generally high for all treatments, indicating a strong antioxidant activity, except for the control drop (CT) and ethephon drop (ED) treatments. The CT and ED exhibited the lowest DPPH inhibition percentage (5.44% and 53.22%, respectively) at the end of the storage period. For the cultivar ‘788’, the DPPH was maintained throughout the whole storage period and there were no significant differences observed between the treatments. Therefore, the ethephon had no significant effect on the DPPH of the ‘788’ harvested nuts. In the case of the cultivar ‘Beaumont’, the CD and ED displayed the highest FRAP values of 8.69 and 8.68 μ moles, respectively on day (Figure 4). Increases in FRAP have been attributed to high TPC and TFC [41]. Therefore, the higher FRAP observed could be a result of the high TPC and TFC observed. After day 0, the ED decreased while the CD maintained the highest FRAP out of all the treatments. On the other hand, for cultivar ‘788’, the CT treatment exhibited the highest FRAP values on day 54 (7.38 μmoles). The ethephon treatment increased from day 0 to 36 and then decreased.

### 3.3 Polyphenol Oxidase Activity

**Figure 5:**
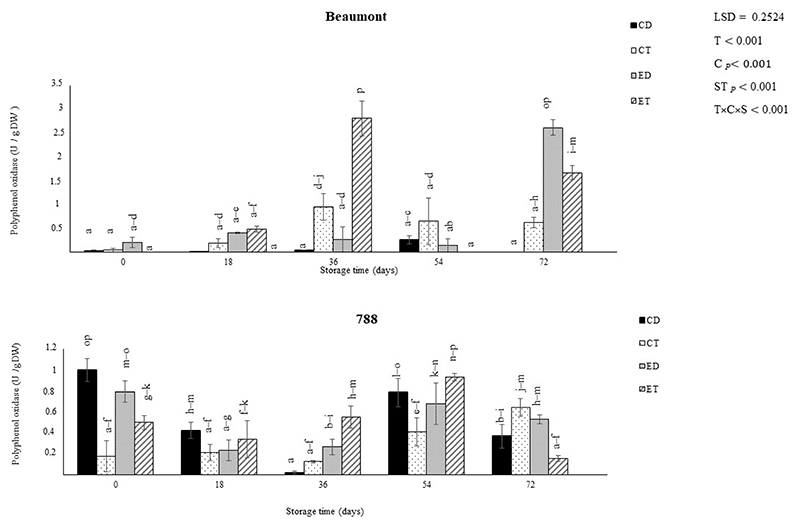
The effect of ethephon application on the polyphenol oxidase activity of macadamia nuts during storage. Data are expressed as mean ± standard errors of three replicates. Vertical bars represented the standard errors of the means. CD represents nuts that dropped from the control tree; CT are nuts that were directly picked from the control tree; ED represents nuts that dropped from the ethephon-treated trees; and ET represents nuts that were picked directly from the ethephon-treated trees. Lowercase letters (a−p) indicate differences between the treatments during storage. T represents treatment (ethephon or control); C stands for cultivar; and S represents storage duration in days.

Polyphenol oxidase (PPO) is an enzyme responsible for the browning of fruits when exposed to the atmosphere or physical abrasions [42]. Figure 5 demonstrates the impact of various treatments (CD, CT, ED, and ET) on the PPO’s activity in macadamia nuts (‘Beaumont’ and ‘788’) stored at 25 °C. In the ‘Beaumont’ cultivar, the control treatments maintained a consistently low PPO activity throughout the storage period, while the ethephon treatments (ED and ET) exhibited the highest PPO activity (1.06 and 0.58 U g^−1^, respectively) at the end of the storage period. Ethephon, which releases ethylene, accelerates senescence in both climacteric and non-climacteric fruits and vegetables [43]. Our findings align with those of [44], who reported that ethephon treatment increases PPO activity and accelerates senescence. For the ‘788’ cultivar, however, the ET and the ED as well as the CD treatment displayed a high PPO activity from the beginning of the storage period, while the CT treatment initially showed the lowest activity (0.18 U g^−1^) on day 0. This indicates a differential response among the two cultivars with the CD treatment being more prone to PPO activity in the ‘788’ cultivar.

### 3.4 Sucrose Content

**Figure 6:**
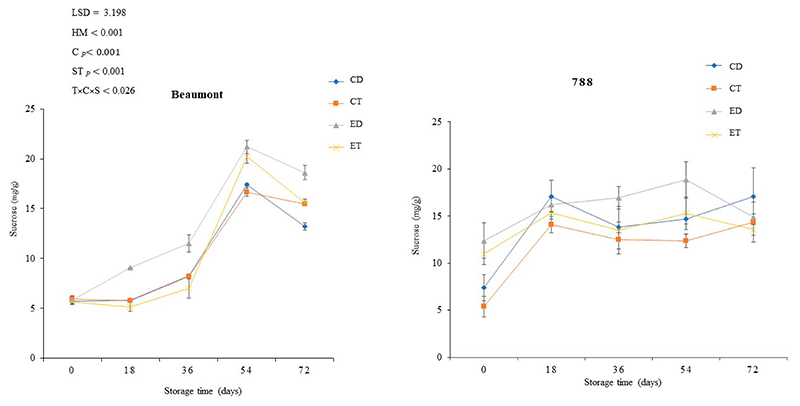
The effect of ethephon application on the sucrose content of macadamia nuts during storage. Data are expressed as mean ± standard errors of three replicates. Vertical bars represent the standard errors of the means. CD represents nuts that dropped from the control tree; CT are nuts that were directly picked from the control tree; ED represents nuts that dropped from the ethephon-treated trees; and ET represents nuts that were picked directly from the ethephon-treated trees. T represents treatment (ethephon or control); C stands for cultivar; and S represents storage duration in days.

Sucrose is the predominant sugar in macadamia nuts, although small amounts of reducing sugars have also been reported [45,46]. The impact of various treatments (CD, CT, ED, and ET) on the sucrose content of macadamia nuts (‘Beaumont’ and ‘788’) stored at 25 °C is illustrated in Figure 6. In the case of the cultivar ‘Beaumont’, the sucrose content increased for all the treatments over the storage period. However, the ED treatment exhibited a more pronounced increase and ultimately had the highest sucrose content (18.63 mg/g) by the end of the storage period. Similarly, in the cultivar ‘788’, the ED treatment displayed a high sucrose (18.84 mg/g) content up to day 54. Additionally, the CT treatment showed a consistently lower sucrose content (5.39–12.35 mg/g) from the beginning of the storage period until day 54. The application of ethephon, a plant growth regulator, can influence the sucrose metabolism in macadamia nuts [47]. Studies have revealed that the presence of sucrose synthase and sucrose-phosphate synthase, which regulate the production of sucrose in plants, can be stimulated through ethephon exposure [47]. This could be the possible reason for the high sucrose content observed in the ethephon-treated nuts for both cultivars. This observation is also similar to the findings of [48], who indicated that sucrose synthase activities were found to be higher in the first 90 days in fruits exposed to ethephon [48]. Also, the variation observed for the different cultivars is similar to the findings of [22], who reported that ethylene treatment increased the stem sucrose content of the low-sugar genotype of sugarcane compared to the high-sugar genotype of sugarcane. The workers further asserted that the sucrose and starch metabolism genes were found to be responsive to ethylene [22].

### 3.5 Total Protein Content

**Figure 7:**
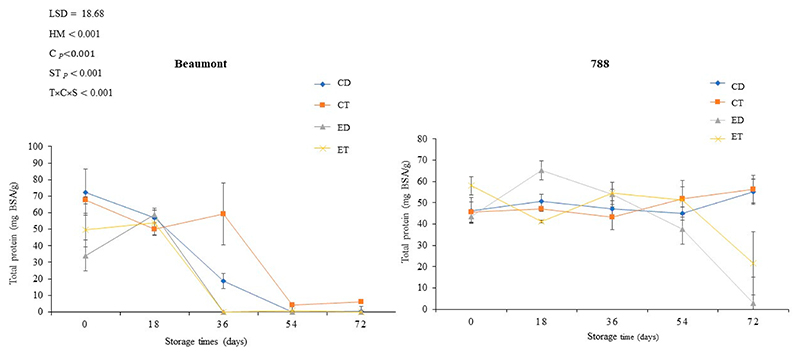
The effect of ethephon application on the total protein content of macadamia nuts during storage. Data are expressed as mean ± standard errors of three replicates. Vertical bars represent the standard errors of the means. CD represents nuts that dropped from the control tree; CT are nuts that were directly picked from the control tree; ED represents nuts that dropped from the ethephon-treated trees; and ET represents nuts that were picked directly from the ethephon-treated trees.

Nuts are a valuable plant-based source of protein, and macadamia nuts typically contain around 8% of protein [49]. The impact of different treatments (CD, CT, ED, and ET) on the total protein content of macadamia nuts (‘Beaumont’ and ‘788’) stored at 25 °C is shown in Figure 7. For the ‘Beaumont’ cultivar, the CD and CT treatments initially exhibited a high protein content (72.4 and 67.5 mg BSA/g, respectively); but, over time, the protein content gradually decreased, reaching 0.5 mg BSA/g for the CD and 6.2 mg BSA/g for the CT by day 72. This decline in protein content can be attributed to the denaturation and solubilization processes that occur at elevated temperatures [50]. Similarly, in the ‘788’ cultivar, the CT and CD treatments showed the highest total protein content (55.2 and 56.4 mg BSA/g, respectively) at the end of the storage period, while the ET and ED treatments had lower protein contents (21.6 mg BSA/g for ET and 2.9 mg BSA/g for ED). In the ‘Beaumont’ cultivar, all the treatments led to a general decrease in the protein content, but the reduction was more pronounced in the ET and ED nuts for both cultivars. This significant decrease in protein content could be attributed to the induced degradation of specific proteins, such as ethylene receptor 2 (ETR2), which occurs at the mRNA (post-transcriptional) level through a proteasome-dependent pathway [51].

### 3.6 Correlation Matrix Heatmap and Principal Component Analysis (PCA)

**Figure 8:**
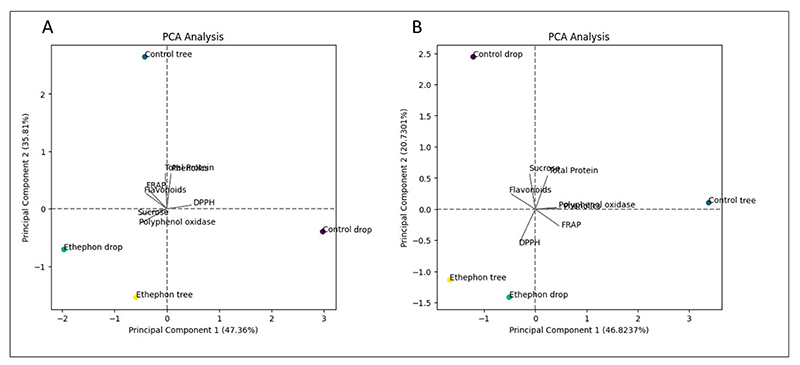
Principle component (PC) biplot of PC1 vs. PC2 showing the relationship between the quality parameters and the different treatments of two macadamia nuts—(**A**) ‘Beaumont’ and (**B**) ‘788’—during storage.

**Figure 9:**
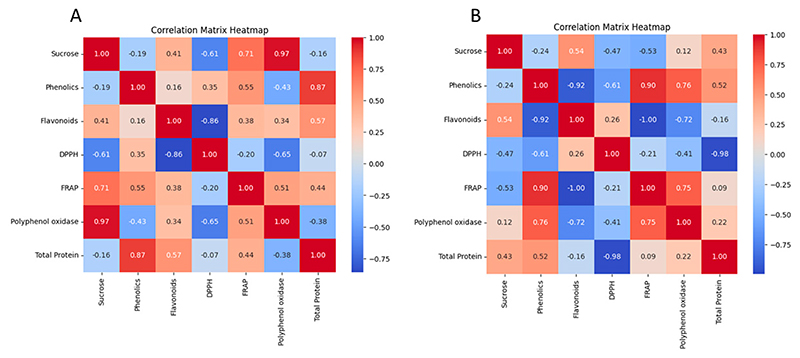
The heatmap representing the correlations between the phenolic contents, flavonoid content, antioxidant activities (FRAP and DPPH), PPO, and total protein during the storage of (**A**) ‘Beaumont’ and (**B**) ‘788’ macadamia nuts. Red and blue colors, respectively, indicate positive and negative correlations; the deeper colors indicate a stronger correlation.

**Figure 10:**
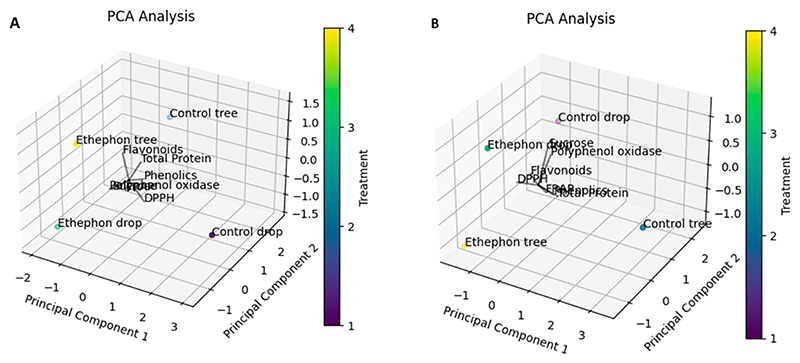
3D Principal component (PC) biplot of PC1 vs. PC2 showing the relationship between the quality parameters and the different treatments of two macadamia nuts—(**A**) ‘Beaumont’ and (**B**) ‘788’—during storage.

The present study conducted an analysis of the principal components of the evaluated biochemical parameters in the ‘Beaumont’ and ‘788’ cultivars using various visualization techniques, including a PCA biplot (Figure 8), a heat map (Figure 9), and PCA 3D plots (Figure 10). The correlation matrix was employed to measure the linear relationship between the parameters, ranging from −1 to +1, where correlations greater than or equal to 0.75 or less than or equal to −0.75 were considered significant. For the ‘Beaumont’ cultivar, the PCA biplot analysis revealed that PCA 1 explained 47.36% of the total variation, while PCA 2 accounted for 35.81% of the variation. Notably, the vectors representing sucrose and PPO were closely aligned in the biplot, indicating a strong positive correlation between these two biochemical parameters. This finding was further supported by the heat map, which displayed a correlation coefficient of 0.97 (Figure 9A). This observation is consistent with the findings of [52], who studied the PPO activity in in vitro rejuvenated and mature shoots of birch. They reported that a high sucrose content was associated with an increased PPO activity. This could be attributed to the sucrose-enhancing phenolic synthesis, which in turn promotes the activity of PPO [52]. Additionally, the directions of the vectors for sucrose and polyphenol oxidase pointed towards the ED treatment, implying a positive correlation with this treatment (Figures 8A and 10A). Similarly, the direction of the total protein vector pointed towards the CT treatment, suggesting a strong correlation with this particular treatment (Figures 8A and 10A). This observation of close proximity and strong correlation was also observed between the total protein and TPC, with a correlation coefficient of 0.87 (Figures 8A and 9A). However, it is important to note that, despite the apparent proximity of the vectors representing the FRAP and flavonoids in the PCA biplot, the PCA 3D plot provided a clearer view indicating the non-proximity and lack of correlation between these two parameters (Figures 8A and 10A). This observation was further supported by the heat map, which revealed a correlation coefficient of 0.38 (Figure 9A). Likewise, a strong negative correlation (−0.87) was observed between the FRAP and DPPH, as confirmed by the heat map (Figure 9A).

For the ‘788’ cultivar, the analysis of the principal components using the PCA biplot indicated that PCA 1 accounted for 46.82% of the total variation, while PCA 2 explained 20.73% of the variation. Notably, the vectors representing the polyphenol oxidase and TPC were closely aligned in the biplot, suggesting a strong correlation between these two parameters (Figure 8B). This observation was further supported by the heat map, which displayed a correlation coefficient of 0.76 for the polyphenol oxidase and TPC parameters (Figure 9B). Phenolic compounds are known to serve as substrates for polyphenol oxidase during the oxidative browning process [53]. Enzymatic browning is the undesirable color change in food caused by the conversion of phenolic compounds that negatively impacts the food’s sensory and nutritional properties [54]. This suggests that the presence of TPC may play a role in activating or influencing the activity of polyphenol oxidase, potentially contributing to the oxidative browning reactions in the macadamia nuts. Furthermore, the vectors of TPC and FRAP were in close proximity to each other, suggesting a strong correlation between these two parameters (Figures 8 and 10). This finding was consistent with the heat map, which revealed a correlation coefficient of 0.9 for the TPC and FRAP parameters (Figure 9). Previous studies have reported a correlation between the TPC and antioxidant activity, as assessed by the FRAP assay [55,56]. Another noteworthy observation was the proximity of the vectors representing the FRAP and polyphenol oxidase (Figure 8). This proximity further suggested a strong correlation between these two parameters, which was supported by the heat map displaying a correlation coefficient of 0.75 (Figure 9). The presence and accumulation of phenolic compounds are closely associated with antioxidant capacity [40]. These findings indicate that the activity of polyphenol oxidase may be influenced by the presence of phenolic compounds, as discussed earlier. Additionally, the alignment of vectors representing the TPC, FRAP, and total protein parameters toward the CT treatment suggests a strong correlation between these parameters and this treatment. This implies that the nuts harvested from the CT treatment exhibit higher levels of total phenolic content, FRAP antioxidant activity, and total protein. However, it is crucial to note that it is not advisable to leave nuts hanging on the tree for several months as it can lead to a deterioration in quality [57].

## 4 Conclusions

Our analysis of the effect of ethephon on the biochemical parameters and polyphenol oxidase (PPO) activity of the ‘Beaumont’ and ‘788’ cultivars of macadamia nuts—harvested through different methods (CT, CD, ET, and ED)—during postharvest storage, revealed significant correlations and associations among the evaluated parameters. In the ‘Beaumont’ cultivar, we observed a strong positive correlation (0.97) between the sucrose and the polyphenol oxidase. Specifically, the nuts treated with the ethephon and harvested from the orchard floor (ED) exhibited the highest sucrose content (18.63 mg/g) and polyphenol oxidase activity (1.06 U) at the end of the storage period. In the ‘788’ cultivar, our analysis demonstrated a close relationship between the phenolics, the polyphenol oxidase (PPO), and the FRAP antioxidant activity. This suggests that the concentration of phenolic compounds in macadamia nuts is closely associated with the activity of polyphenol oxidase, an enzyme involved in browning reactions, as well as the overall antioxidant capacity measured by FRAP. The strong association between these factors indicates that the phenolics present in the nuts may contribute to the oxidative browning process and play a role in their antioxidant activity. Considering these findings, the CT treatment emerges as the most favorable option as it consistently showed positive correlations with multiple key parameters in both cultivars. This suggests that nuts harvested using the CT method may have a longer shelf life due to their higher phenolic content and antioxidant activity. However, it is important to acknowledge that the CT treatment, which involves harvesting nuts directly from the tree, can be labor-intensive. In contrast, the ethephon treatment is easier to implement but may not yield the same level of desired outcomes. Therefore, for future considerations, it would be beneficial to focus on optimizing the biochemical parameters and minimizing the polyphenol oxidase (PPO) activity of nuts harvested from ethephon-treated trees in order to improve their quality during storage. Further research efforts can explore strategies to enhance the shelf life and maintain the nutritional quality of nuts treated with ethephon, potentially bridging the gap between the convenience of implementation and the desirable outcomes. Further investigations can explore strategies to enhance the shelf life and preserve the nutritional value of nuts treated with ethephon, considering factors such as storage conditions, postharvest treatments, and processing techniques.

## Data Availability

Data are contained within the article.
